# Thank you Reviewers

**DOI:** 10.1177/2325958218775471

**Published:** 2018-07-09

On behalf of the editorial office, we would like to express our deepest gratitude to the reviewers who took the time to provide us with invaluable feedback on manuscripts submitted to *Journal of the International Association of Providers of AIDS Care*. Without their support and commitment, we simply could not publish the journal.

Adoga, Moses
Ajiboye, Justus
Akinyemi, Joshua
Altaf, Arshad
Antunes, Francisco
Arogundade, Linda
Atkins, S.
Bastos, Joao Luiz
Beck, Stefan
Berentsen, Sigbjorn
Blanco, Jose Ramon
Branson, Bernard
Browning, Kristine K.
Bruce, Douglas
Chang, Larry
Christian, Chandler
Colasanti, Jonathan
Collins, Alexandra
Cotte, Laurent
Crane, Larry
daneshfar, Fatemeh
Demetrovics, Zsolt
Di Biagio, Antonio
Dionne-Odom, Jodie
Doro Altan, Annamaria
Edokwe, Chidozie
Ekong, Ernest
Eriobu, Nnakelu
Estrada Perez, Vicente
Etienne-Mesubi, Martine
Evangeli, Michael
Fair, Cynthia
Feldman, Matthew
Fierer, Daniel
Galato, Dayani
Gerle, Elisabeth
Giordano, Tom
Greenberg, Ken
Grzeszczuk, Anna
Hanna, David
Hodgson, Ian
Holstad, Marcia
Horberg, Michael
Imashuku, Shinsaku
Jacomet, Christine
Joglekar, Alok
Kaplan, Rachel
Karris, Maile Y.
Keshavamurthy, Vinay
Kietrys, David
Kikuchi, Kimiyo
Kingori, Caroline
Kulkarni, Vaman
Kumar, Ganesh
Lam-ubol, Aroonwan
Laws, Michael Barton
Lee, Cliff
Lewis, Joseph
Liao, Bing
Magidson, Jessica
Makinson, Alain
Marhefka, Stephanie
Matthews, Lynn
Mehrjerdi, Zahra
Menzaghi, Barbara
Muliira, Joshua
Muralidharan, Shrikanth
Murphy, Richard A.
Musa, Vera
Mwiru, Ramadhani
Myer, Landon
Neogi, Ujjwal
Ngure, Kenneth
Nozza, Silvia
Nwankwo, Norbert
Ogoina, Dimie
Okada, Seiji
Özakgül, A. A.
Pacek, Lauren
Pereira, Marco
Phanuphak, Nittaya
Philbin, Morgan M.
Pirak, Arezoo
Popoola, Oladayo
Poudel, Krishna
Rajagopal, Jahnavi
Rana, Aadia
Rebeiro, Peter
Reddy, V. R. Sudha
Rodriguez-Diaz, Carlos
Rutledge, Scott
Sabin, Caroline
Sanders, E. J.
Sawers, Larry
Shetty, Avinash
Short, William
Sigel, Keith
Silva-Vergara, Mario Leon
Singh, Vijendra
Slim, Jihad
Sonderup, Mark
Soriano, Vincent
Sterrantino, Gaetana
Strehlau, R.
Swendeman, Dallas
Umeh, Clifford
Vance, David
Walker, Naomi
Wortley, Pascale
Young, Benjamin

